# Transforming a Negotiation Framework to Resolve Conflicts among Older Adults and Family Caregivers

**DOI:** 10.3390/geriatrics8020036

**Published:** 2023-03-08

**Authors:** Alaine Murawski, Vanessa Ramirez-Zohfeld, Allison Schierer, Charles Olvera, Johnathan Mell, Jonathan Gratch, Jeanne Brett, Lee A. Lindquist

**Affiliations:** 1 Division of Geriatrics, Feinberg School of Medicine, Northwestern University, Chicago, IL 60208, USA; 2 School of Computer Science, University of Central Florida, Orlando, FL 32816, USA; 3 Institute of Creative Technologies, University of Southern California, Los Angeles, CA 90007, USA; 4 Kellogg School of Business, Northwestern University, Evanston, IL 60208, USA

**Keywords:** family caregivers, negotiation, conflict resolution, Alzheimer’s dementia

## Abstract

Background: Family caregivers of older people with Alzheimer’s dementia (PWD) often need to advocate and resolve health-related conflicts (e.g., determining treatment necessity, billing errors, and home health extensions). As they deal with these health system conflicts, family caregivers experience unnecessary frustration, anxiety, and stress. The goal of this research was to apply a negotiation framework to resolve real-world family caregiver–older adult conflicts. Methods: We convened an interdisciplinary team of national community-based family caregivers, social workers, geriatricians, and negotiation experts (*n* = 9; Illinois, Florida, New York, and California) to examine the applicability of negotiation and conflict management frameworks to three older adult–caregiver conflicts (i.e., caregiver–older adult, caregiver–provider, and caregiver–caregiver). The panel of caregivers provided scenarios and dialogue describing conflicts they experienced in these three settings. A qualitative analysis was then performed grouping the responses into a framework matrix. Results: Upon presenting the three conflicts to the caregivers, 96 responses (caregiver–senior), 75 responses (caregiver–caregiver), and 80 responses (caregiver–provider) were generated. A thematic analysis showed that the statements and responses fit the interest–rights–power (IRP) negotiation framework. Discussion: The interests–rights–power (IRP) framework, used in business negotiations, provided insight into how caregivers experienced conflict with older adults, providers, and other caregivers. Future research is needed to examine applying the IRP framework in the training of caregivers of older people with Alzheimer’s dementia.

## 1. Introduction

Over 44 million family or informal caregivers provide unpaid assistance and support to older people and adults with disabilities who are living in the community [1]. A family caregiver is defined as someone who provides unpaid care to a relative or friend, who is 18 years or older, to help them take care of themselves [2]. Increasingly, family caregivers are taking care of more than one person at the same time, many of whom have Alzheimer’s disease [3,4].

Alzheimer’s disease (AD) poses unique challenges to family caregivers [5]. Currently, an estimated 6.5 million Americans have been diagnosed with Alzheimer’s disease, and this is expected to rise to 12.7 million by 2050 [6]. Nearly 80 percent of people with Alzheimer’s dementia (PWD) receive help with activities of daily living compared to just 20 percent of older adults without dementia [7,8]. Over time, a PWD is not able to fully understand medical instructions or independently communicate with their health care team [9,10]. As a result, many older adults with AD rely on family caregivers to function on a day-to-day basis and complete health-related tasks [7,8,9,10]. In addition to their Alzheimer’s disease diagnosis, PWD are more likely than the general older population to experience complex comorbidities (e.g., diabetes, heart failure, and renal failure), multiple providers, and polypharmacy [11]. Thus, while they are engaged in supporting the PWD in daily personal activities, such as bathing, dressing, and feeding, caregivers often facilitate the medical care, including scheduling/accompanying to appointments, assisting with medications, dealing with insurers, and communicating with members of the health care team [12].

Prior research has shown that family caregivers act as “helicopter advocates” or patient navigators for their PWD, interacting with the health system during conflicts, e.g., determining if a test/procedure/medication/hospitalization is truly necessary, responding to insurance denials or billing errors, home health extensions, and scheduling issues [13]. We surveyed a national cohort of 97 family caregivers using open-ended questions inquiring about experiences with the healthcare system and constant comparative qualitative analysis of responses. Family caregivers discussed extensive conflicts with physicians, nurses, and insurance companies, including having to confirm and clarify medications, calling for new supplies, and examining options for procedures. These interactions and conflicts with the healthcare system were frustrating, stressful, time consuming, and time wasting [14]. Family caregivers of PWD have also reported a lack of communication by healthcare providers, leading to complications concerning medications and, ultimately, frustration and a lack of trust in their loved one’s healthcare provider [15,16]. A reoccurring theme from this prior research demonstrates that family caregivers experience frustration, anxiety, and stress as they deal with these health system conflicts. Whether it is spending hours on the phone trying to obtain answers from a health care provider’s team or navigating complex insurance policies, these conflicts contribute to the burden experienced by a family caregiver [13,14,15,16].

Caregivers also navigate conflicts with older adults and other family members [17,18]. A large conflict between caregivers-older adults occurs when the older adult refuses additional services in the home (e.g., paid caregiver assistance and physical therapy) [19]. Our previous research has shown that this conflict arises from the older adult feeling a loss of independence, attempting to avoid burdening others, and a lack of trust in other supporters [20].

Another area of conflict is with other family members (e.g., caregiver siblings and PWD’s spouse/partner) [21]. A frequent conflict in this context is between the caregiver who has first-hand knowledge of the needs of the older adult and family members who are not involved in the day-to-day care and do not agree with adding support: “My sister doesn’t think mom (who has AD) is that bad so she’ll drop her off at Target and then yell at her when she can’t find her. I tell her don’t leave her alone but then she does!” Additionally, siblings have differing opinions on home care options: “My brother and his wife want to put mom in a nursing home because she’s so far gone (with AD). But, I could never do that to my mom” [19]. Sex differences exist in these family caregiver negotiations, with male caregivers less likely to resolve disagreements in favor of the patients’ preferences [22,23].

All of these types of conflicts can increase the frustration, anxiety, stress, and burden for the family caregiver [24,25]. To decrease these negative outcomes experienced during conflicts, family caregivers need to have the tools to resolve these conflicts. Family caregivers often lack formal training in conflict resolution. While there is a long history of research on negotiation and conflict resolution and negotiation and conflict management training for managers and lawyers, which has been available since the early 1980s, this knowledge and training have not been available or adapted for family caregivers. Currently, there is a gap in how negotiation and conflict dispute resolution training programs can be framed and tailored to family caregivers. Doing so is extremely important because research in the negotiation context shows that people have difficulty transferring learning from one context to another [26,27]. This project is the first part of a larger project to develop and assess the utility of a conflict management training program for family caregivers. In this research, we gather caregivers’ conflict management experiences and assess the fit between their experiences and conflict management theory.

## 2. Materials and Methods

An interdisciplinary team composed of negotiation experts, a social worker, geriatrician, and a community-based family caregiver panel (*n* = 9; Illinois, Florida, New York, and California) was convened. As professors and faculty teachers of negotiation at large academic business schools, the negotiation experts used an iterative approach to critically review the frameworks that are taught in business and law school negotiation and dispute resolution courses [28]. The main features sought in a framework was ease of use, skills required, adaptability, and relevance. The interdisciplinary team was then convened, and the negotiation experts provided options and recommendations for a framework to be used to organize the conflict management experiences of family caregivers of PWD.

Concurrently, the caregiver panel, consisting of community-based family caregivers from Florida, Illinois, and New York, convened to develop scenarios of family caregiver attempts to manage family caregiver conflicts involving a caregiver–older adult (CA), caregiver–caregiver (CC), and caregiver–healthcare provider (CH). To create the scenarios, the caregiver panel members were asked to write a short scenario of when they had a disagreement with an older adult, family caregiver, or healthcare provider, respectively.

Using the scenarios, the family caregiver panel created scripted dialogues that would realistically occur between the two participants in the conflict scenario. The dialogue was broken into statements and responses—with multiple statement–responses being possible depending on the complexity and length of the scenario. Once the scenarios and corresponding dialogues were compiled, they were coded into the proposed conflict management framework by two coders using constant comparative analysis [28]. Using an iterative triangulation approach [29], the scenarios, corresponding dialogues, and coding were then reviewed by the interdisciplinary team to determine the fit between the caregiver conflict management experience and the conflict management framework. The Northwestern University Institutional Review Board considered this work to be exempt.

## 3. Results

### 3.1. Deciding on a Negotiation Framework

#### 3.1.1. Deal Making versus Dispute Resolution

Negotiations occur in many contexts; however, two major ones are deal making and dispute resolution. Deal making is transactional. Parties are deciding on the compensation for goods or services that one is providing to the other. In this context, prior to negotiating, the parties have identified each other as potentially the best possible partner with whom to conduct the transaction. If they cannot reach an agreement, each can negotiate with another provider. Deal-making negotiators may become emotional but, at least to begin with, their emotions should be positive. In contrast, dispute resolution is relational. Typically, one party has made a claim and the other has rejected that claim. For example, in the caregiver–older adult context, the caregiver may tell the older adult, “I’m arranging for housecleaning”. The older adult may reject that offer, “I take care of my house, I don’t need any help”. In dispute resolution, emotions are engaged before the negotiation even begins. The caregiver is frustrated that the plan was rejected, the older adult does not want change. The challenge, then, is how to bring these opposing interests together, which tuns into a negotiation to minimize costs, rather than maximize gains, as in deal making. Finally, if the disputing parties fail to reach an agreement, the problem is still there. Whereas in deal making, the parties turn to negotiate with other providers of the good or service [26].

Three structural elements of negotiation are different in dispute resolution and deal making: negotiating emotions, minimizing costs versus maximizing gains, and the implications of no agreement.

The strategies, or goal-directed behaviors, that negotiators can use in the two contexts are similar. Negotiators can identify and reconcile interests, or they can use influence to try to get the other party to concede. In fact, deal making and dispute resolution negotiators use both strategies, even in the same negotiation. The strategy of identifying and reconciling interests is essentially the same in dispute resolution as it is in deal making negotiations. However, in dispute resolution, rights-based arguments, particularly references to fairness, past practice, and even contract or law, play a larger role than is typical of deal-making negotiations. The rejection of an offer that leads to a dispute is often justified with rights or power. “I’m not spending my money to have a cleaning woman come and break everything in my house” is a power statement that nevertheless reveals interests. The challenge for the negotiator is to redirect the other party from rights or power to interests.

#### 3.1.2. The Interest–Rights–Power (IRP) Framework

The interdisciplinary team chose the IRP dispute resolution framework rather than the deal-making negotiation framework recognizing that the conflicts that caregivers become involved in as helicopter advocates have more of the structural elements of dispute resolution than deal-making negotiations. The interests–rights–power (IRP) framework describes the three types of statements that are commonly made in a negotiation. Interest statements show a person’s reasoning for taking a position in a negotiation. Rights statements argue the legitimacy of a person’s position in a negotiation. Power statements reveal a person’s authority over the other person in a negotiation [30].

Negotiators often use all three types of statements to try to resolve conflict. However, the type of resolution implied by the use of interests versus rights or power statements is different. Conceding to a rights or power statement means giving in. The result of the negotiation is a winner and a loser. Conceding to an interest statement is different. Doing so implies that both parties’ interests and concerns are met. There is no single winner and loser. To encourage the resolution of conflict, the IRP framework encourages redirecting negotiators focused on rights or power statements to interests [31].

In addition, research on the IRP framework has identified five ways to redirect rights- or power-focused negotiators to interests. These are:

1. Do not reciprocate the rights or power statement. Instead, make an interests-based suggestion.
2. Do not become personal and blame the other party for the problem.
3. Reciprocate the rights or power argument but follow up directly with an interests-based suggestion.
4. Propose a process intervention. Suggest putting the argument aside and brainstorming an interests-based solution.
5. Suggest following the advice of a trusted third party.

The IRP framework and the five ways to redirect rights- or power-focused negotiators to interests provides a conceptual platform for organizing the experiences of caregivers negotiating with an older adult (CA), caregiver–caregiver (CC), and caregiver–healthcare provider (CH) [24].

### 3.2. Family Caregiver Conflict Scenario and Dialogue Development

Caregivers generated the following number of lines of dialogue for the conflicts: CA (*n* = 96), CC (*n* = 75), and CH (*n* = 80). Two coders working with the theoretical definitions of IRP and the five ways to redirect rights and power to interests, iteratively developed a code book of caregiver examples using a small subset of scenarios from each caregiver context. Then, the coders assigned each line of dialogue in each scenario to a codebook category or to the “other” category. In this way, the coding of each scenario revealed movement through interest, rights, and power.

To show movement through interest, rights, and power, in the coding of the dialogue an initial statement was added in front of the offer statement. Statements were created using the IRP framework based on the experiences of family caregivers’ conflict between a family caregiver and older adult, family caregiver and family caregiver, and family caregiver and health care provider.

The codebook identified interest statements as those used to learn about the overall “why” of each person’s position in the conflict. For example, the statement “What do you think we should do moving forward?” is an interest statement because it is asking a question to try to arrive at the other person’s “why”. On the other hand, the statement “It’s my responsibility to make sure you safe” is a rights statement because it is addressing the caregiver’s responsibility to ensure the older adult is safe. An example of a power statement is, “You don’t know what you’re talking about”. This statement would be considered an attack.

### 3.3. Applying the IRP Framework to Caregiver Dialogue

The coding resulted in a matrix of data for each dialogue. The matrix revealed the focus of each statement in the dialogue but also the movement between statements as the negotiators moved toward agreement or impasse. An example of a scenario created by a family caregiver on the caregiver panel is below. This scenario describes conflict between two family members over the care of their older adult loved one:

*Once a week, a home health nurse assisted my elderly father who has dementia, mobility impairment due to a stroke, and is immunosuppressed due to a series of organ transplants. My mother and adult sister are the primary caregivers in my parents’ home, and they had a disagreement regarding the nurse’s visits. One of the nurse’s weekly tasks was to assist my dad with a bandage/gauze change for a nasty bedsore he’d developed from a previous hospital stay. This task was something that both my sister and the nurse performed regularly. My mother did not want the nurse to come into the home at all, however my sister appreciated the nurse’s help. My sister asked me to step in and agree with her against my mother that they should keep the help.*

As you can see from the above, this scenario shows themes of conflict with a family member over the level of care their older adult loved one should receive. Below is an example of a conflict scenario between two siblings over increasing the care in the home of the older adult.

*You are the primary caregiver for your parents. Your sibling is not as involved in your parent’s care. You feel that your parents need more care in the home. They recently have had health issues arise that have made it difficult for them to take care of themselves. However, your sibling disagrees with you. You want your sibling to agree for more care/support for your parents; and You want their help convincing your parents to agree to more care/support.*

In addition, below is an example of the resulting dialogue that was generated by the caregiver panel and coded into the IRP framework (Table 1).

## 4. Discussion

Conflicts frequently occur as family caregivers advocate for the health of their PWD. This research showed that interests–rights–power (IRP) provides a useful framework to organize the dialogue that occurs when family caregivers negotiate conflict. The evidence generated by this research—that conflict dialogues experienced by caregivers fit with an existing conflict management framework—provides a strong basis for building a negotiation training for caregivers that is grounded in theory and practice.

In addition, there is relatively little real-world negotiation research on conflict situations that generate emotions, such as those described by caregivers in this study [32,33]. Thus, this research provides support for the original conceptualization of interest, rights, and power as an approach to resolving disputes in the context of disputes between labor and management to an entirely different context caregiver conflict [27]. This research also provides support for the original research that identified different patterns of movement among interests, rights, and power that ultimately break into reciprocity of rights or power statements that lead to impasses [29].

This research showed that the IRP framework is relevant to the dialogues that caregivers have when they are in conflict with older adults and others involved in their care. Our experience in coding the conflict dialogues shows that once the fundamental concepts are understood, it is easy to identify the use of interests, rights, and power statements and to see how these dialogues move between discussions of interests to influence attempts based on rights or power, and if the caregiver is creative in continuing to return to interests, to agreement.

Thus, the research provides a theoretical and practical basis for our next step, which is to develop a program to train family caregivers, most of whom likely have no formal training in negotiation, to use interests, rights, and power to resolve conflicts. Further plans will then be to determine if a training program built on the IRP framework is effective in teaching caregivers how to navigate conflicts throughout the caring process.

Due to the current lack of formal training in negotiation and conflict management, family caregivers might not intuitively be able to identify interest, rights, or power statements in everyday conversation. The question for future research is if family caregivers can learn the interest–rights–power framework and apply it to their conflict situations. One limitation is cultural adaptation, beyond translation. The dialogues developed in this research may require adaptation for training family caregivers in cultures where norms for confrontation, or reliance on social norms, for example, may be different. For example, blaming and shaming may be more prevalent in non-Western culture as is reliance on powerful third parties [22]. Of course, IRP is not the only negotiation framework that could be used to train family caregivers, and there may be alternative options. The interdisciplinary panel considered other applications, including the classic integrative distributive negotiation framework [25], as well as the conflict styles framework [26]. However, the analysis of the interdisciplinary team and the data provided by the caregivers panel suggest that the IRP framework has the characteristics of ease of use, skills required, adaptability, and relevance, which are necessary for building a family caregivers conflict management training program.

## 5. Conclusions

In conclusion, the interests–rights–power (IRP) framework provides insight into how family caregivers go about trying to resolve conflicts with older adults, providers, and other caregivers. The framework also provides a tool to examine the interests, rights, and power of the involved parties (e.g., family caregivers, older adults, and providers) and understand the outcomes of their attempts to negotiate the resolution of conflict. Coding the scenarios using the IRP framework provides examples of how strategic movement leads to agreement or impasse. Future research will examine the application of the IRP framework in the negotiation training of family caregivers of older adults.

## Figures and Tables

**Table 1:** Resulting dialogue.

Sibling Caregiver #1	Sibling Caregiver #2	IRP
I don’t see what the big deal is. I think Mom and Dad are fine. (Power)	You’re kidding, right? Stop being so ridiculous!	Power
	I’m around a lot more than you and I see it.	Rights
	Can we please try to work together on this?	Interests
What? You’re being selfish. (Power)	It would help if they think we are both on the same page.	Interests
This all seems like a lot of work and confrontation, and all for an hour of help? (Rights)	I don’t mind helping every day—but I am more comfortable knowing we have people to help.	Rights
It may be easier to adjust slowly now, rather than us deal with finding a caregiver when something bad happens. (Interest)	We both want the best for them. Let’s try just a bit of outside help to start.	Interests

## Data Availability

Not applicable.
